# Co_3_O_4_/Al-ZnO Nano-composites: Gas Sensing Properties

**DOI:** 10.3390/s19040760

**Published:** 2019-02-13

**Authors:** Ada Fort, Enza Panzardi, Valerio Vignoli, Mokhtar Hjiri, Mohamed Salah Aida, Marco Mugnaini, Tommaso Addabbo

**Affiliations:** 1 Department of Information Engineering and Mathematical Sciences, University of Siena, 53100 Siena, Italy; panzardi@diism.unisi.it (E.P.); valerio.vignoli@unisi.it (V.V.); marco.mugnaini@unisi.it (M.M.); addabbo@diism.unisi.it (T.A.); 2 Department of Physics, Faculty of Sciences, King Abdulaziz University, Jeddah 21589, Saudi Arabia; m.hjiri@yahoo.fr (M.H.); aida_salah2@yahoo.fr (M.S.A.)

**Keywords:** nano-materials, gas sensors, hetero-junctions, metal oxides sensors, chemoresistors

## Abstract

In this paper, the gas sensing properties of metal oxide nano-powder composites are studied and modeled. The gas sensing properties of mixtures of two different metal oxide nanoparticles, prepared via low-cost routes, are investigated. The responses to both an oxidizing (NO_2_) and a reducing gas (CO) are analyzed. The tested composites are obtained by mixing a different percentage of a p-type metal oxide, Co_3_O_4_, with moderate responses to NO_2_ at about 200 °C and to CO at high temperature (above 260 °C), with n-type Al-doped ZnO, which is characterized by a large but unstable response towards NO_2_ around 160 °C and a moderate response towards CO around 200 °C. In the oxides mixtures, p-n heterojunctions are formed by the juxtaposition of an n-type and a p-type grain in contact. Consequently, the electronic conductivity is modified and the obtained composite materials show novel characteristics with respect to the base materials. This indicates that predicting the behavior of the composites from those of their components is not possible and it suggests that the hetero-junction behavior has to be studied to understand the sensing properties of the composite materials. The obtained results indicate that the composites containing a significant amount of hetero-junctions exhibit a stable response to NO_2_ at room temperature and significant responses towards CO at 160 °C.

## 1. Introduction

Today, the research has shown that using nano-structured metal oxides can enhance the conductometric gas sensor performance and partly solve or mitigate many of their problems. Experiments and subsequent theoretical simulations [1] have shown that, in general, nano-materials are particularly promising for the development of gas sensors [2,3,4,5,6,7,8,9,10], since surface-related properties that are important for gas sensing are amplified in the nanoscale when the surface/volume ratio is very large. 

A further development in the field is pursued through the application of composite materials.

Actually, generally speaking, the idea of using composite material to improve the performance of conductometric metal-oxide gas sensors has been largely applied. In fact, the addition of noble metals, such as platinum or gold, through the impregnation or incorporation in the oxide matrix, is an old one, and its effectiveness has been proven by a huge amount of literature and for many different metal oxides. More recently, the addition of nano-particles or of nano-clusters of noble metal has been considered [11,12].

Besides the traditional sensing materials that were obtained by combining metal-oxides with noble metals, many other composites have been proposed and studied, which are synthetized by mixing a metal oxide with a polymer, carbon nanotubes, graphene, or, finally, with a different metal oxide. In particular, many researches have focused on this last type of complex materials (Me^I^O/Me^II^O), establishing that significant improvements in terms of sensitivity, selectivity, stability, and power consumption can be obtained [3,4,7,9,13,14,15,16,17,18,19,20,21,22,23,24,25,26,27]. An extensive review on this topic can be found in Ref. [28].

There are a great number of methods that are used for metal oxide/metal oxide nano-composites preparation [29,30,31,32,33,34,35,36,37,38]. The most facile route is mixing the already-synthesized materials in given proportions, milling, and then subsequently heating. This method allows for certainty in that the material properties of the two base components (Me^I^O and Me^II^O) are the same, even when preparing different mixtures with different proportions of the two oxides. On the other hand, with other preparation methods, such as impregnation or co-precipitation, the simultaneous presence of the two precursors in the starting solution could give rise to modifications of the structure of the two base materials and it is not easy to understand the final material structure: beyond pure Me^I^O and Me^II^O domains, different phases can appear. For example, during co-precipitation, the incorporation of Me^II^ into the lattice of Me^I^O or vice versa is possible, especially when trying to form a composite with a low concentration of one of the two base oxides, causing either bulk doping of the most abundant oxide or formation of solid solutions. As a rule, a separated second phase Me^II^O appears in the Me^I^O matrix at the additive concentration exceeding the solubility limit of Me^II^ in Me^I^O. Moreover, spinel phases, such as Me^I^_x_Me^II^_y_O_z_, can be also formed. An interpretation of the results that were obtained while studying sensors, fabricated using such methods is very difficult because the sensing materials can undergo unpredictable modifications depending on the two metals relative concentrations, needing a complex characterization of each individual composite. Mixing the already synthetized nano-materials overcomes these problems. 

Note that metal oxides that are used for conductometric gas sensing are semiconductors, and their response to gases exploits the dependence of the electronic conduction on the electronic exchange between the gas phase and the solid material (through chemisorption). Hence, as widely discussed in the literature, the gas sensing properties of metal oxides are related to many different aspects, which can be detailed, as follows:

1. The bulk electronic structure of the metal oxide, which determines the electronic properties and hence the electronic conduction.
2. The film morphology and structure: the size and the shape of the grains forming the film are particularly important in establishing the sensitivity of the sensor. The electronic conduction in ceramic, in fact, involves free carriers moving through the bulk of each grain, but also across the boundaries of the neighbor grains.
3. The surface reactions. Obviously, the basic process that determines the sensor response is the chemisorption (adsorption and ionization of a gas molecule). Since the surface defect population is usually one of the two reactants (the other is the gas), the surface activity also depends on the specific preparation route. Moreover, the surface activity toward a given gas depends heavily on the possible presence of catalysts and, in composite materials, one of the two components can act as a catalyst and activate/favor some specific reaction on the surface of the other component. The catalytic effect is one of the explanations for the enhanced sensitivity and response speed or the reduced operating temperature of metal oxides that are doped with noble metals.

Summarizing the gas sensing properties of a composite that was prepared by mixing two already-synthetized metal oxides cannot be derived simply from the properties of the two base materials. However, as discussed in points 2) and 3), it is controlled by the formation of hetero-junctions in the mixture, and by the possible catalytic role that one material can play for chemisorption on the other.

In this paper, we analyze the gas sensing properties of nano-grained composites that were obtained by mixing the nano-powders of Co_3_O_4_ (a p-type semiconductor) and of Al-doped ZnO (a n-type semiconductor) in different proportions, with a particular focus on the influence of the p-n hetero-junctions that were formed by these two materials. The behavior of heterojunctions of ZnO and Co_3_O_4_ was studied in Ref. [39] as a light emitting structure. Moreover, different gas sensing materials embedding this type of junctions, as obtained by different routes, have been proposed in the literature for the detection of formaldehyde, ethanol, and NO_2_, but the most part of these materials consist of heterostructure where one of the material surrounds a core of the second and is the one that is exposed to gases [40,41,42,43,44], therefore the heterojunctions participate in the electronic conductions but could be not directly affected by the gas adsorption. On the other hand, the proposed materials exploit the direct effect of gas adsorption at the heterojunction barriers. The gas sensing behavior of the proposed materials is analyzed and compared to one of the base materials exploiting the results that were obtained with mixtures of air and CO or NO_2_, also in the presence of humidity. 

## 2. Materials and Methods

### 2.1. Instrumentation for Material Characterization

The microstructure of the samples was investigated by XRD (Bruker AXS D8 Advance) using the CuKα1 wavelength of 1.5405 Å. The average crystallite size was calculated while using the Scherrer’s formula:

(1) $$G=\frac{0.9\lambda }{B\cos\theta_{B}}$$

where *λ* is the X-ray wavelength, *θ_B_* is the maximum of the Bragg diffraction peak (in radians), and B is the full width at half maximum (FWHM) of the XRD peak. 

The grain shape and size of the prepared powders were monitored by means of transmission electron microscopy (TEM) that was carried out with a JEOL JEM 2010 electron microscope (LaB6 electron gun, JEOL(ITALIA), Milan, Italy) operating at 200 kV and equipped with a Gatan 794 Multi-Scan CCD (Gatan GmbH, München, Germany) camera for digital imaging. The samples were prepared by placing a drop of the samples dispersed in isopropanol on 400 mesh holey-carbon coated copper grids.

### 2.2. Gas Sensing Properties Characterization System

In this work, the experimental data were collected through the measurement system that was described in Refs. [45,46] and developed to simultaneously characterize up to eight sensors. The sensors are placed in a circular array exploiting eight front-end boards hosting the conditioning and acquisition electronics mounted on a main board and that are placed in a steel measurement chamber. The system provides an accurate measurement of the gas sensing film temperature, with an uncertainty that is lower than 3 °C for temperatures in the range [120 °C, 400 °C].

The sensors were tested in the temperature range of 120 °C–260 °C, in the presence of two different toxic gases, CO and NO_2_, mixed with synthetic air (20% oxygen and 80% nitrogen), with an RH value of 0% and 50% at 25 °C.

All of the presented measurements are obtained by applying a specific protocol and by repeating each measurement three times. The applied protocol consists in exposing the sensor to a constant flow of 200 mL/min; each measurement consists of 4 min in a flow of synthetic air (carrier gas), 4 min in a mixture of air and CO or NO_2_, and 8 min again in air to allow for the recovery of the surface. Different concentration values were used for tests ranging from 6 to 2000 ppm for CO and from 6 to 50 ppm for NO_2_.

The variation of the electrical resistance of the chemoresistive gas sensor is the sensor output; the sensor response is defined as:

(2) $$Response=\frac{R-R_{0}}{R_{0}}$$

In Equation (2), *R*_0_ is the baseline resistance value in the carrier gas (air), whereas *R* is the resistance value after a fixed duration exposure to a target gas at a given concentration.

## 3. Materials Preparation and Characterization

### 3.1. Synthesis of ZnO-Al5% Nanoparticles

The sol-gel technique was used to prepare ZnO: Al5% nanopowders using 16 g of zinc acetate dehydrate [Zn(CH_3_COO)_2_ × 2H_2_O; 99%] as a precursor in 112 mL of methanol. After 10 min of magnetic stirring at room temperature, an adequate quantity of aluminum nitrate-9-hydrate corresponding to [Al]/[Zn] ratio of 0.05 were added. After 15 min under magnetic stirring, the solution was placed in an autoclave and then dried at 250 °C according to protocol that is reported in Ref. [46]. Subsequently, the obtained powder was annealed at 400 °C for 2 h.

### 3.2. Preparation of Co_3_O_4_ Nanoparticles

Co_3_O_4_ nanoparticles were synthesized by hydrothermal route. The precursor material was Co(NO_3_)_2_·6H_2_O powder. This precursor was dissolved in water after magnetic stirring. Afterwards, some drops of ammonia were added to the solution. The final solution was poured into a Teflon lined steel autoclave, which was kept for heating in a programmed furnace at 200 °C for 10 h. The resultant material was washed several times with mixture of ethanol and water for purification and then dried at 60 °C for 1 h in an oven. Subsequently, the obtained powder was annealed for 2 h at 500 °C.

**Figure 1:**
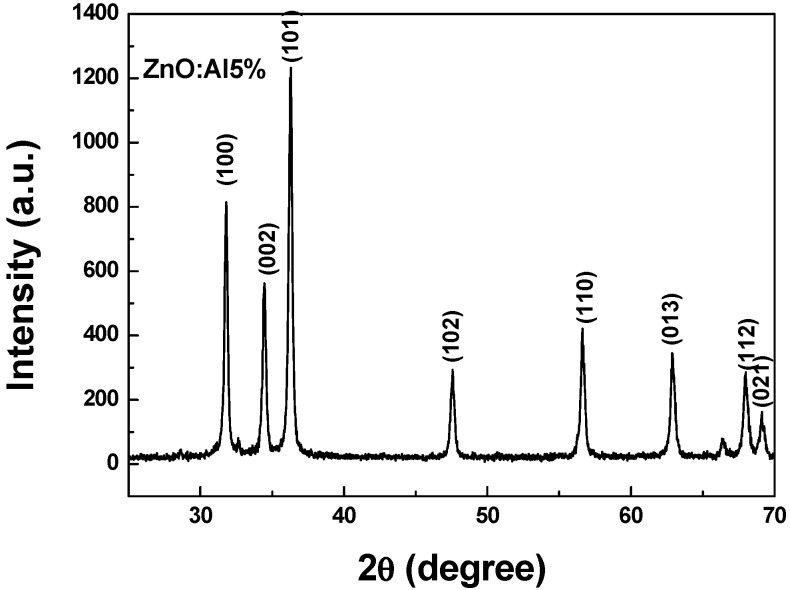
X-ray diffraction spectrum of ZnO: Al5% sample.

**Figure 2:**
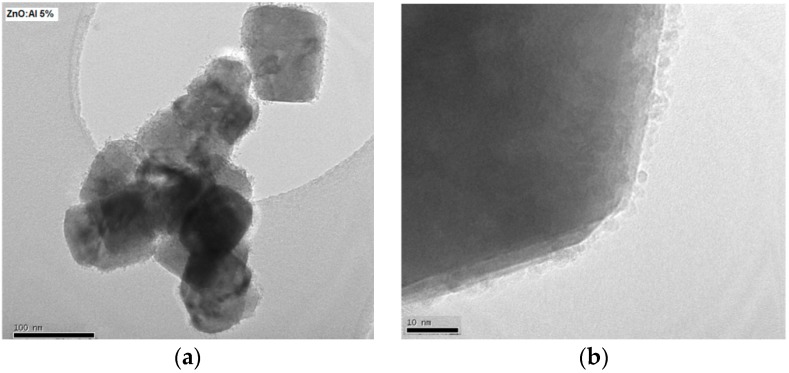
(**a**) Transmission electron microscopy (TEM) and (**b**) High Resolution TEM (HRTEM) images of Al-doped ZnO nanoparticles.

Figure 1 shows the X-ray diffraction patterns that were registered for the Al-doped ZnO sample annealed at 400 °C for 2 h in air, indicating that the sample is polycrystalline. The pattern of ZnO sample shows peaks corresponding to (100), (002), (101), (102), (110), (103), and (112) planes. All above mentioned diffraction peaks are assigned to the Wurtzite hexagonal-shaped ZnO with space group P63mc (JCPDS card N° 36–1451) [10]. No extra peaks were registered, indicating that this sample is composed of single phase ZnO. The average ZnO crystallite size has been estimated to be 63 nm. However, small crystallites of Al are observed in the TEM image reported in Figure 2; due to their small sizes, they can’t be detected by the XRD technique.

**Figure 3:**
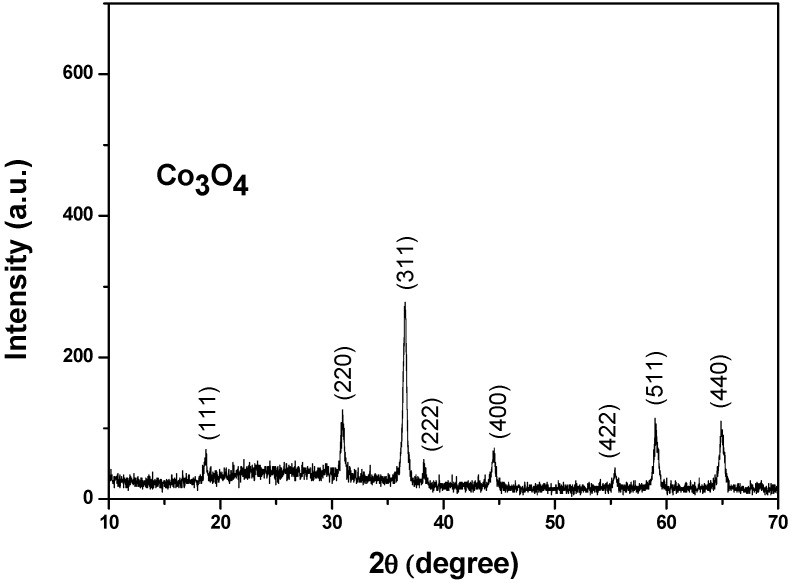
X-ray diffraction of Co_3_O_4_ nanoparticles.

The TEM image reported in Figure 2 shows the shape of the grains. The measured ZnO crystallite size is about 63 nm. In addition, we note the presence of smaller nanoparticles, having a size below 5 nm, as shown in Figure 2b. This secondary structure is likely due to a heterogeneous nucleation and the successive growth of Al crystallite on the surface of ZnO particles already formed. Previous research indicated the formation of a ZnAl_2_O_4_ spinel phase that is rich in Al detected at high Al content [47]. In our case, this phase was not detected in the XRD pattern, indicating that Al atoms are distributed in a ZnO network and they may act as dopant. Figure 3 presents the X-ray diffraction pattern of Co_3_O_4_ nanoparticles. All of the reflection peaks are assigned to (111), (220), (311), (222), (400), (422), (511), and (440) diffraction planes of Co_3_O_4_ cubic phase (JCPDS Card file No. 74–1656) [48]. There are no extra peaks indicating the purity of the material. The average crystallite size that was calculated from Scherer’s equation has been estimated to be 60 nm.

**Figure 4:**
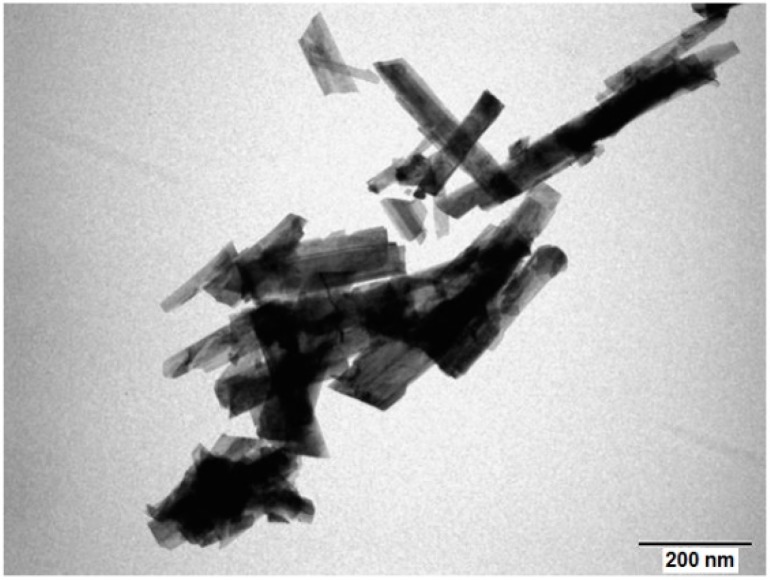
TEM images of Co_3_O_4_ nanoparticles.

The TEM image of the prepared sample is given in Figure 4. The particles have a regular elongated shape, with a width of about 60 nm and more than 200 nm of length. 

## 4. Sensor Preparation

In order to test all of the prepared composites, some sensors were realized [38,46] on ad hoc alumina substrate (size 8 mm × 15 mm × 0.26 mm of thickness) with pre-deposited electrodes for the sensing film, a heater on the backside, and a Pt-based Resistance Temperature Detector (RTD), with all being deposited by screen printing. The realization of a RTD Pt-based sensor close to the gas sensing film allows an accurate measurement of the operating film average temperature, which is of utmost importance for the accuracy of the gas sensor characterization. 

The conductor material for electrodes is based on Ag/Pt, which utilizes an oxide bond system for providing adhesion to alumina oxide substrates.

The nano-powders of the two metal oxides, prepared as described in the previous section, were hand mixed and milled at room temperature, adding ultra-pure water, and obtaining a homogeneous paste. The sensing layer is obtained by drop-coating the region across the two electrodes using a micro-pipette. After the deposition, the device was heated at 350 °C for 24 h. 

## 5. Gas Sensing Modeling

Spinel structure Co_3_O_4_ is a mixed valence oxide of CoO and Co_2_O_3_. Therein, Co^2+^ and Co^3+^ ions are located at the tetrahedral 8a sites and the octahedral 16d sites, respectively. Part of the two ions charge can exchange between the two sites, hence stoichiometric Co_3_O_4_ also behaves as a p-type semiconductor showing that the majority carriers are free holes. The material is characterized by a bandgap energy 2.04 eV [49]. It has catalytic properties and the adsorbed active oxygen species were observed by XPS analysis. Nano-structures of this material have been obtained by many different preparation techniques and subsequently proposed as gas sensing material for different oxidizing and reducing gas (a review can be found in Xu et al. [50]). Recently, it was shown that chains of nano-particles (70 nm diameter) that were obtained by a one-step thermal treatment method could be used for NO_2_ detection at room temperature (response of 52%to 100 ppm NO_2_), but the response and recovery times are rather long [51].

ZnO is a material that finds a lot of sensing applications, and for this reason, it has been extensively studied. The material is characterized by wide band-gap energy (3.15–3.4 eV), such that the intrinsic carrier density at room temperature is of the order of 10^6^ cm^−3^. ZnO behaves as n-type material, due to native defects, most probably impurities ([52,53]), or less favored interstitial zinc atoms or oxygen vacancies, with an intrinsic donor level being located 0.51–0.17 eV below the conduction band [54]. The donor density at room temperature is typically in the order of 10^16^–10^17^ cm^−3^. The electrons mobility is about 200 cm^2^/(Vs), whereas the holes mobility is in the range of 5–50 cm^2^/(Vs). Al doped ZnO has larger band-gap energy, in the range (3.32–3.77 eV) [54], with a donor level being located at 0.12 eV below the conduction band. ZnO has shown great potential for sensing toxic and combustible gases, such as CO, H_2_, NH_3_, C_2_H_5_OH, and H_2_S. 

**Figure 5:**
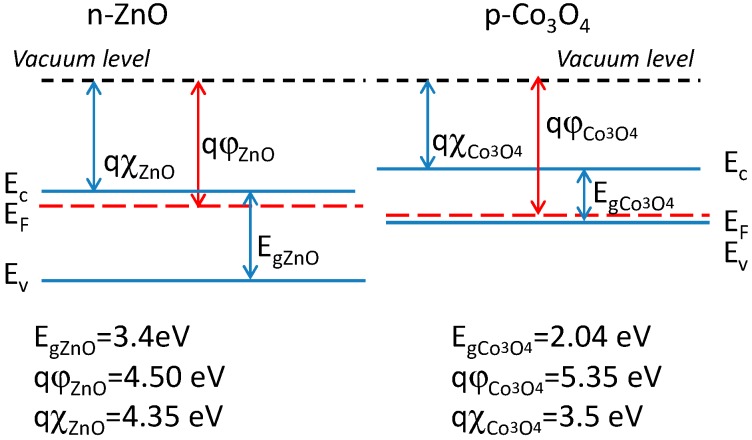
Electronic band diagram for the two materials. Numeric data from [39] qφ indicates the work function and qχ the electronic affinity, whereas Eg is the band-gap energy.

A schematic description of the electronic band configuration of the two materials is shown in Figure 5.

The resistance of each sensing film can be modeled as a parallel of *k* resistances, *R_path_*, connecting the two electrodes, each resistance *R_path_* is made up of small adjacent nano-particles. On average, the series resistances can be considered to be equal since the current path is formed by a large number, *M*, of particles:

(3) $$M=\frac{l_{path}}{\Phi }\approx \frac{0.3\;\mathrm{mm}}{70\;\mathrm{nm}}\approx 4300$$

where $l_{path}$ is the mean length of the percolation path and Φ is the average particle size. Moreover, a similar distribution of p-type particles and n-type particles can be considered for each current path. In conclusion, the film resistance is approximately equal to *R_path_*/*k*, where *R_path_* represents a percolation path, and it consists of the summation of the resistive contribution of the particle bulks, and of the surface regions of adjacent grains in contact and *k* is the number of current paths in the film. At the grain boundaries, in the studied material, two types of homo-junctions (p-p and n-n) and p-n heterojunctions can be formed. At the homojunctions, Schottky barriers are formed, which give the most relevant contribution to *R_path_*, whereas, at the p-n junctions a hetero-junction is formed.

In summary, we can write:

(4) $$R_{path}\approx \sum R_{ZnO/ZnO}+\sum R_{Co_{3}O_{4}/Co_{3}O_{4}}+\sum R_{Co_{3}O_{4}/ZnO}$$

where $R_{ZnO/ZnO}$ ($R_{Co_{3}O_{4}/Co_{3}O_{4}}$) represents the resistance of the Schottky barrier between two ZnO (Co_3_O_4_) nanoparticles, whereas $R_{Co_{3}O_{4}/ZnO}$ is the resistance of the junction between Co_3_O_4_ and ZnO particles.

**Figure 6:**
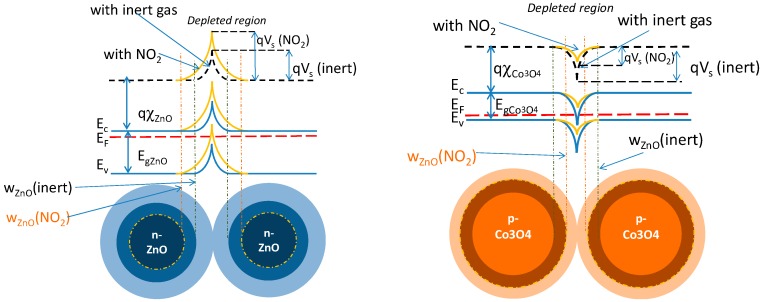
Schottky barrier formation and the effect of an oxidizing gas.

The Schottky barriers originate from the accumulation of trapped charges on the metal oxide grains surface, due to the ionization of intrinsic surface defects and to the adsorbed species. See in Figure 6.

The resistance describing the Schottky barrier (considering small applied external voltage), where the current is due to thermionic emission, can be written as:

(5) $$R_{ZnO/ZnO}=R_{A_{ZnO}}\;e^{\frac{qV_{s}{}_{ZnO}}{k_{B}T}}$$

(6) $$R_{Co_{3}O_{4}/Co_{3}O_{4}}=R_{A_{Co_{3}O_{4}}}\;e^{\frac{qV_{s}{}_{Co_{3}O_{4}}}{k_{B}T}}$$

where $R_{A_{ZnO}}\left(R_{A_{Co_{3}O_{4}}}\right)$ is a resistance that is weakly dependent on temperature and independent of the gas effect; it can be considered a constant, $k_{B}$ is the Boltzmann constant, *q* is the charge of the electron, *T* is the absolute temperature, whereas $V_{s_{Co_{3}O_{4}}}\;\mathrm{and}\;V_{s_{ZnO}}$ are the voltage barriers at the two homojunctions. Equations (5) and (6) describe a temperature activated conduction where $R_{A_{ZnO}}\left(R_{A_{Co_{3}O_{4}}}\right)$ is the pre-exponential coefficient, whereas $qV_{s_{ZnO}}=E_{AZnO}$ ($qV_{s_{Co_{3}O_{4}}}=E_{s_{Co_{3}O_{4}}}$) represent the activation energies. The gas sensitivity is due to the changes of this barrier height due to variations of the charge trapped at the surface through the creation of the so-called ‘extrinsic surface states’, i.e., allowed energy state localized at the surface that can trap free electrons if empty (case of an acceptor or oxidizing gas) or donate electrons if occupied (gas of a donor or reducing gas). We can better specify this relationship writing:

(7) $$V_{s_{Co_{3}O_{4}}}=f\left(N_{s_{Co_{3}O_{4}}}\right);\;V_{s_{ZnO}}=f\prime \left(N_{s_{ZnO}}\right)$$

The equations state that the barrier height is a function of *N_s_* ($N_{s_{Co_{3}O_{4}}},\;N_{s_{ZnO}}$), which is the surface density of the ionized adsorbed molecules (chemisorbed).

Starting from the assumption that a surface is natively depleted of the majority carriers, i.e., assuming intrinsic surface defects of donor type for the p-type material, and for the n-type material, instead, of acceptor type, then we can write:

(8a) $$\mathrm{for}\;p-\mathrm{type}:\;N_{s_{Co_{3}O_{4}}}=N_{i_{Co_{3}O_{4}}}+{\left[S_{x}-X\right]}^{+}-{\left[S_{y}-Y\right]}^{-}$$

(8b) $$\mathrm{for}\;n-\mathrm{type}:\;N_{s_{ZnO}}=N_{i_{ZnO}}-{\left[S_{x}-X\right]}^{+}+{\left[S_{y}-Y\right]}^{-}$$

where [Ξ] indicates that the surface concentration of the species Ξ, *S_X_* (*S_Y_*) are adsorption sites for the *X* (*Y*) species (from the gaseous phase), and finally $N_{i_{ZnO}}\left(N_{i_{Co_{3}O_{4}}}\right)$ denotes the surface density of the intrinsic ionized defects. In this paper, we consider that *X* corresponds to CO or water vapor and *Y* to NO_2_.

The shape of the function *f* (*f*’) depends on the geometry of the grains [1,12], for large grains it is well known that:

(9a) $$\mathrm{for}\;p-\mathrm{material}:\;V_{s}=\frac{qN_{s}^{2}}{2\epsilon p_{b}}$$

(9b) $$\mathrm{for}\;n-\mathrm{material}:\;V_{s}=\frac{qN_{s}^{2}}{2\epsilon n_{b}}$$

where *n_b_* and *p_b_* are the bulk carrier densities (usually considered constant in the working temperature range and, being *N_A_* (*N_D_*) the bulk density of acceptors (donors), *n_b_* ≈ *N_D_* and *p_b_* ≈ *N_A_*). In the case of nano-structures, this equation can only give a coarse approximation of *f*, but, in any case, it can still describe the main characteristics of the sensor behavior [1].

**Figure 7:**
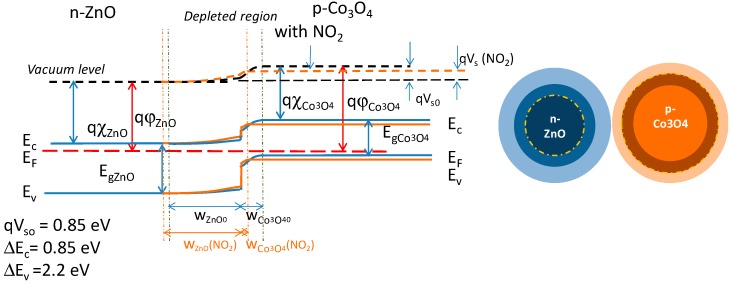
Formation of the heterojunction and behavior with an oxidizing gas.

In the case of two different material particles in contact, then the heterojunction is formed, as shown in Figure 7. In this figure, the effect of an oxidizing chemisorbed gas is also depicted.

For the studied heterojunction in the absence of trapped surface charges, a depleted region is formed at the barrier due to free electrons diffusion in the p-region, whereas free holes diffuse in the n-type one. The built-in barrier height is $qV_{s}=q\left(\phi_{Co_{3}O_{4}}-\phi_{ZnO}\right)$. The widths of the two depleted regions, $w_{0}{}_{ZnO}$ and $w_{0}{}_{Co_{3}O_{4}}$, are related to the electronic characteristics of the two materials as:

(10) $$w_{0_{ZnO}}=\sqrt{\frac{2\epsilon_{ZnO}}{qn_{b}}V_{sn}};\;w_{0_{Co_{3}O_{4}}}=\sqrt{\frac{2\epsilon_{Co_{3}O_{4}}}{qp_{b}}V_{sp}}$$

where $V_{sn}$($V_{sp}$) the voltage barrier in the p (n) material. The current density is described by drift-diffusion as in a normal p-n junction and it can be written as:

(11) $$J_{ZnO/Co_{3}O_{4}}=\left(\frac{qD_{eCo_{3}O_{4}}}{\Phi_{Co_{3}O_{4}}}\frac{n_{i}{}_{Co_{3}O_{4}}^{2}}{p_{b}}+\frac{qD_{h_{ZnO}}}{\Phi_{ZnO}}\frac{n_{i}{}_{ZnO}^{2}}{n_{b}}\right)\left(e^{\frac{qV_{e}}{k_{B}T}}-1\right)\approx \frac{qD_{eCo_{3}O_{4}}}{\Phi_{Co_{3}O_{4}}}\frac{n_{i}{}_{Co_{3}O_{4}}^{2}}{p_{b}}\left(e^{\frac{qV_{e}}{k_{B}T}}-1\right)$$

where $D_{eCo_{3}O_{4}}$ $\left(D_{hZnO}\right)$ is the diffusion coefficient for electrons (holes), $\Phi_{Co_{3}O_{4}}$ ($\Phi_{ZnO}$) is the diameter of the Co_3_O_4_ (ZnO) grain, $n_{i}{}_{Co_{3}O_{4}}$ $(n_{i}{}_{ZnO}$) is the intrinsic carrier density. Finally, *V_e_* is the applied external voltage. Note that, as shown by the last term of the equation, due to the smaller bandgap, most probably the contribution to the current of minority electrons from the p-material dominates. In the presence of a gas or in general regarding trapped charge on the surface, the two depleted regions are modified, for instance, for the ZnO grain, we can write:

(12) $$w_{ZnO}=w_{ZnO}{}_{0}\pm \Delta w_{ZnO}$$

For electroneutrality:

(13) $$\Delta w_{ZnO}=\frac{\Delta N_{s}{}_{ZnO}}{n_{b}}$$

This equation shows the relationship between the chemisorbed species density and the junction behavior.

Similar equations can be written for the Co_3_O_4_ grain. For instance, in Figure 7, the effect of an oxidizing gas is shown, which consists of electrons trapping or holes generation, causing the narrowing of the depleted region in the p-material and its widening in the n-material. Consequently, the built-in voltage changes, so that: $V_{s}=V_{s0}+\Delta V_{s}$. This variation can be modelled as an external field perturbing the equilibrium and modifying the current intensity. As such, at the end, when considering a small external applied field and linearizing the relationship between current and applied voltage, we can write:

(14) $$R_{ZnO/Co_{3}O_{4}}\approx \frac{c\;e^{\frac{q\Delta V_{s}}{k_{B}T}}}{\frac{qD_{eCo_{3}O_{4}}}{{\;\Phi }_{Co_{3}O_{4}}}\frac{n_{i}{}_{Co_{3}O_{4}}^{2}}{p_{b}}}=R_{A_{ZnO/Co_{3}O_{4}}}\;e^{\frac{E_{A_{ZnO/Co_{3}O_{4}}}}{k_{B}T}}$$

where c is a coefficient depending on the geometry. Equation (14), as Equations (5) and (6), describes a temperature activated conduction where $R_{A_{ZnO/Co_{3}O_{4}}}$ is the pre-exponential coefficient, whereas $E_{A_{ZnO/Co_{3}O_{4}}}$ represents the activation energy, which depends, as discussed in this section, on the charge that is trapped on the surface by the adsorbates through the term Δ*V_s_*. This relationship explains the gas sensing mechanism and indicates a behavior that is similar to the one of the homojunctions. As such, the sensitivity to a specific target gas is given by:

(15) $$\begin{array}{c} \frac{dR_{ZnO/Co_{3}O_{4}}\;}{d{\left[X\right]}_{gas}}=\frac{dR_{ZnO/Co_{3}O_{4}}\;}{d\Delta V_{s}}\left(\frac{d\Delta V_{sn}}{dN_{s}{}_{ZnO}}\;\frac{dN_{s}{}_{ZnO}}{d{\left[X\right]}_{gas}}+\frac{d\Delta V_{sp}}{dN_{s}{}_{Co_{3}O_{4}}}\frac{dN_{s}{}_{Co_{3}O_{4}}}{d{\left[X\right]}_{gas}}\right)=\\ q\frac{R_{ZnO/Co_{3}O_{4}}}{k_{B}T}\;\left(\frac{d\Delta V_{sn}}{dN_{s}{}_{ZnO}}\;\frac{dN_{s}{}_{ZnO}}{d{\left[X\right]}_{gas}}+\frac{d\Delta V_{sp}}{dN_{s}{}_{Co_{3}O_{4}}}\frac{dN_{s}{}_{Co_{3}O_{4}}}{d{\left[X\right]}_{gas}}\right) \end{array}$$

where [*X*]*_gas_* is the concentration of the target gas, whose absorption originates from a variation of $N_{s}{}_{ZnO}$ and $N_{s}{}_{Co_{3}O_{4}}$, according to Equation (8a) and (8b). The magnitude of the sensitivity depends on many factors, in particular, the electronic characteristics of the two materials determining the variation of *V_sn_* and *V_sp_*, given a specific density of adsorbed charge [1] and the chemical affinity of the two materials toward the target gas, which can also be tuned by the possible catalytic effect of one of the two material with respect to the other.

Note that the sign of $\Delta V_{s}$ depends on the characteristics of the materials and on their affinity to a given gas, because $\Delta V_{s}=\Delta V_{sn}+\Delta V_{sp}.$ As an example, in Figure 7 it is shown that an oxidizing gas causes a positive $\Delta V_{sn}$ and a negative $\Delta V_{sp}$.

## 6. Experimental Results 

### 6.1. Resistance Baseline 

**Figure 8:**
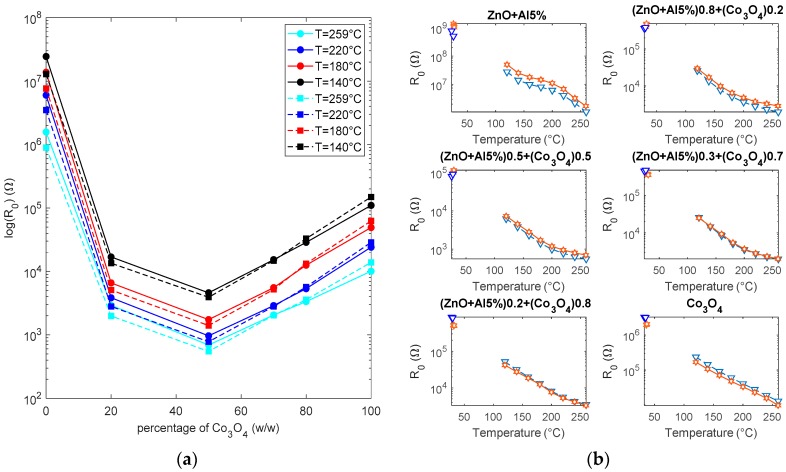
(**a**) Baseline resistance as a function of the percentage of Co_3_O_4_ in the mixtures (w/w). Continuous lines—dry air. Dashed lines—humid air (RH = 50%). (**b**) Baseline resistance as a function of temperature for the different mixtures. Hexagon markers—dry air, triangle markers—humid air (RH = 50%).

**Figure 9:**
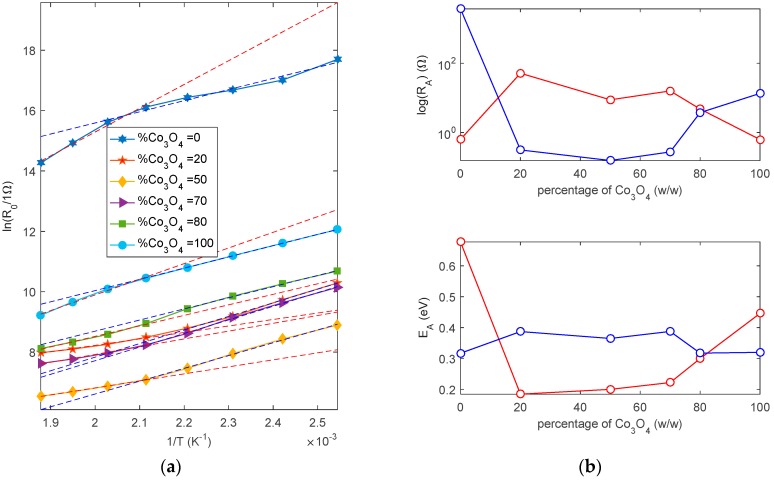
(**a**) ln(R_0_) vs. 1/T, markers-experimental values, dashed red lines-linear fitting in the high temperature range (200 °C–260 °C), blue dashed lines—linear fitting in the low temperature range (120 °C–200 °C). (**b**) Estimated parameters E_A_, R_A_ from the two fittings (see Equations (5), (6) and (14)). Linear fitting performed according to the following relationship $\ln\left(R_{0}\right)=\ln\left(R_{A}\right)+\frac{E_{A}}{k_{B}}\frac{1}{T}$. Red lines—linear fitting in the high temperature range (200 °C–260 °C), blue lines—linear fitting in the low temperature range (120 °C–200 °C).

The baseline resistance was studied both in dry and in humid air. In particular, in Figure 8a, the baseline resistance versus the content of C_3_O_4_ is plotted: the effect of the heterojunctions formation can be clearly seen. In fact, the mixture with a 50% Co_3_O_4_ content presents a baseline resistance in air that is lower than the one of the two base materials, indicating that the heterojunction barrier is lower than both the Schottky barriers. Moreover, as expected, for the humidity increases the resistance for the n-type material (ZnO) and on the opposite decreases the resistance for the p-type material. Moreover, water vapor increases the resistance for mixtures with Co_3_O_4_ content up to 70%, whereas for mixtures with a higher concentration of Co_3_O_4_, the resistance decreases in the presence of humidity. The mixture that is characterized by 70% Co_3_O_4_ has a baseline that is almost insensitive to humidity. In Figure 8b, the dependence of the baseline resistance on temperature in air is plotted for the different materials. In Figure 9, the base line resistances in dry air are fitted using Equations (5), (6), and (14), and when considering a constant value for the vs. (ln(*R*_0_) vs. 1/*T* is linearly fitted) in two temperature ranges up to 200 °C and above 200 °C. The values of *E_A_* and *R_A_* (see Equations (5), (6), and (14)) derived from linear regression for low and high temperatures are shown in Figure 9b. The fitting is accurate only if, in the presence of oxygen, the barrier height is almost constant in the considered temperature range, i.e., *N_s_* is constant in this experimental condition. It can be seen that, for Co_3_O_4_, the approximation is quite good, showing that the intrinsic and oxygen related surface ionized charge have an almost constant density in the two ranges of the temperature. The estimated “activation energy” (*E_A_*) values are 0.38 eV (low T) and 0.45 eV (high T). For ZnO, the barrier height varies, which is probably due to the significant oxygen chemisorption; the value of the activation energy is lower for low temperature (0.33 eV) and larger at high temperature (0.7 eV). The mixtures with a Co_3_O_4_ content lower than 80% show a variation of the barrier height at temperatures above 200 °C that could be interpreted as the effect of ionized adsorbed oxygen reducing the barrier heights. 

### 6.2. Response to NO_2_

The response of the sensor to dry and humid mixture of air and NO_2_ are presented and discussed in this section.

The surface reaction, which is usually considered to be responsible for the behavior of p-type and n-type metal oxide sensors, is the following [12]:

(16a) $$NO_{2}+S_{NO_{2}}+e^{-}\Leftrightarrow \;{\left(S_{NO_{2}}-NO_{2}\right)}^{-}\;\;(n-\mathrm{type})$$

(16b) $$NO_{2}+S_{NO_{2}}\Leftrightarrow \;{\left(S_{NO_{2}}-NO_{2}\right)}^{-}+h^{+}\;\;(p-\mathrm{type})$$

**Figure 10:**
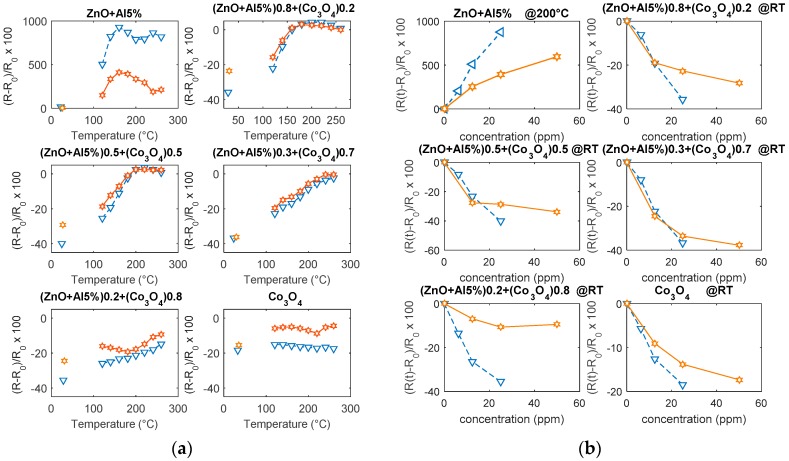
(**a**) Response to 25 ppm of NO_2_ as a function of temperature. Marker triangles—humid environment (RH = 50%). Marker stars—dry environment. (**b**) Sensor response vs. NO_2_ concentration at 200 °C (ZnO-Al5%) and room temperature (RT) (the other materials).

As it can be seen in Figure 10, ZnO-Al(5%) has a very large sensitivity toward NO_2_, especially at low temperatures (15%/ppm @120 °C @25 ppm), but the response transients are so slow at temperatures lower than 200 °C that the use of this sensor is impractical. In fact, at 200 °C, the response is still satisfactory and the response time (10%–90%) decreases to about 1 min (@25 ppm NO_2_), whereas the recovery time is approximately 2 min in dry mixtures. Note that, below this temperature, the sensor response, as defined in Equation (2), is an underestimation of the actual one since the sensor does not reach the steady state during gas exposure and does not fully recover. Moreover, the response is dramatically influenced by humidity: the response increases (almost doubles), as does the speed. At 200 °C, for instance, the response time drops to 0.7 min (@25 ppm NO_2_) and recovery time becomes about 1.5 min. The response of Co_3_O_4_ is small in the tested temperature range. The transient behavior appears complex, being characterized by a fast initial transient, followed by a slow decrease of the resistance, suggesting that the surface reaction in Equation (16) is not sufficient to describe the response. In the presence of humidity, the response shows a large increase, and the time behavior changes, suggesting the existence of an interaction between the adsorbed OH^−^ and H^+^ and target gas. 

**Figure 11:**
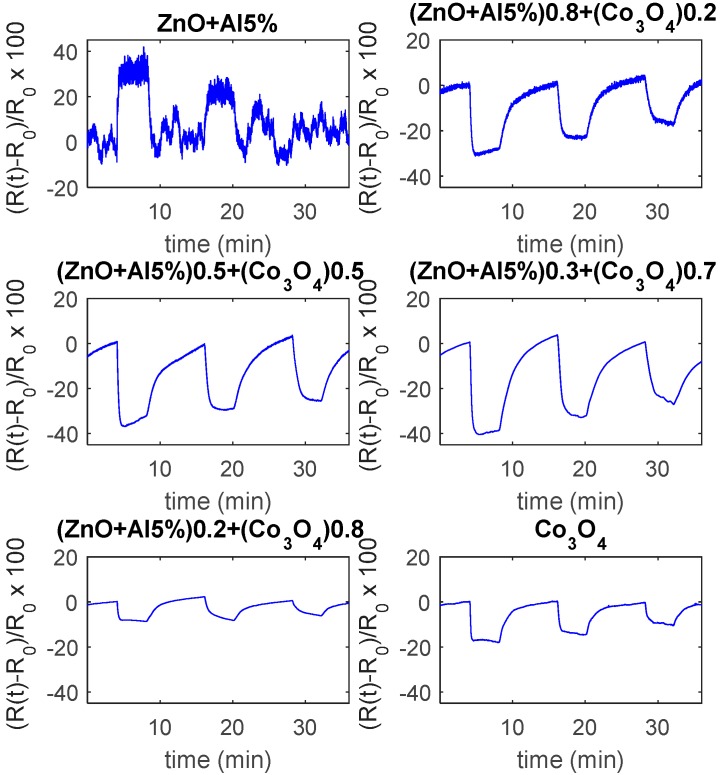
Response to variable NO_2_ concentrations as a function of time, at room temperature (RT), for the tested materials. The measurements are performed changing the test mixture composition in the following way: 4 min dry air, 4 min dry air and NO_2_ (50 ppm), 8 min dry air, 4 min dry air and NO_2_ (25 ppm), 8 min dry air, 4 min dry air and NO_2_ (12.5 ppm), 4 min dry air.

All of the composites respond with a decrease of the resistance to the target gas and they are characterized by fast responses, even at room temperature, the slow decrease that was observed for Co_3_O_4_ disappears. In particular, the composites that were obtained with a 70% Co_3_O_4_ have a fast and satisfactory response at a low temperature (even at room temperature 1.5%/ppm @25 ppm) and its magnitude is almost insensitive to humidity. As it can be seen from the results shown in Figure 11, the response speed is satisfactory with a response time, for dry mixtures, at room temperature, of 0.5 min (@25 ppm NO_2_ and a recovery time of about 5 min).

### 6.3. Response to CO

The response to CO was studied both in the dry and humid environment. 

The reactions, which are usually considered at the basis of CO sensing for metal oxide sensors, are the following [55,56,57,58]:

(17a) $$CO+S_{CO}\Leftrightarrow \;{\left(S_{CO}-CO\right)}^{+}+e^{-}\;\;(n-\mathrm{type}\;\mathrm{material})$$

(17b) $$CO+S_{CO}+h^{+}\Leftrightarrow \;{\left(S_{CO}-CO\right)}^{+}\;\;(p-\mathrm{type}\;\mathrm{material})$$

(18a) $$CO+{\left(S_{o}-O\right)}^{-}\Rightarrow S_{O}+CO_{2}+e^{-}\;\;(n-\mathrm{type}\;\mathrm{material})$$

(18b) $$CO+{\left(S_{o}-O\right)}^{-}+h^{+}\Rightarrow \;S_{O}+CO_{2}\;\;(p-\mathrm{type}\;\mathrm{material})$$

Reactions (17a) and (17b) are direct (and reversible) chemisorptions at the material surface, whereas reactions (18a) and (18b) involve pre-adsorbed oxygen and they are considered not reversible.

**Figure 12:**
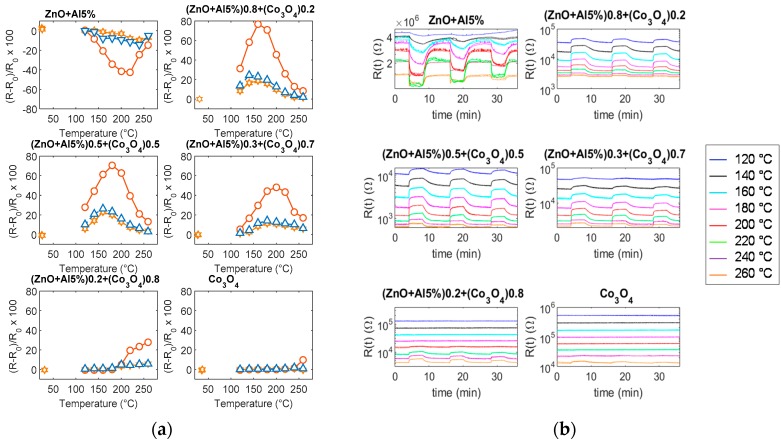
(**a**) Response to CO as a function of temperature. Marker triangles—humid environment, air and 25 ppm CO (RH = 50%). Marker stars—dry environment, air and 25 ppm CO. Marker circles—dry environment and 1000 ppm CO. (**b**) Responses to variable CO concentrations as functions of time for the different materials. The eight graphs in each subplot are the sensor responses recorded at different and constant temperatures in the range (120 °C–260 °C) with 20 °C steps, as per legend. The measurements are performed changing the test mixture composition in the following way: 4 min dry air, 4 min dry air and CO (2000 ppm), 8 min dry air, 4 min dry air and CO (1000 ppm), 8 min dry air, 4 min dry air and CO (500 ppm), 4 min dry air.

**Figure 13:**
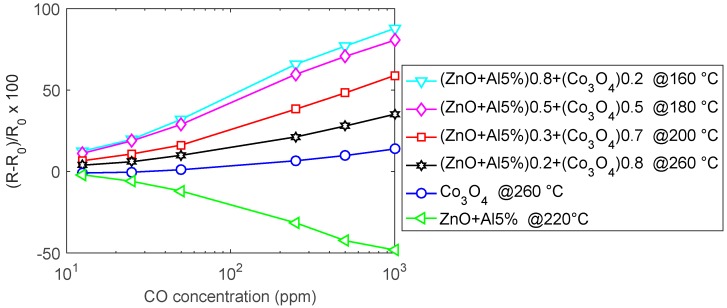
Response to CO as a function of CO concentration (dry environment).

As it can be seen in Figure 12, the pure ZnO-Al5% has a satisfactory response to CO above 160 °C, the maximum sensitivity is reached at 200 °C/220 °C, above 200 °C the response is very fast but of a lower amount, and it is characterized by instability (Figure 12b). The appearance of instabilities and oscillations, especially for high concentrations of CO, is probably related to the formation of CO_2_ in the presence of pre-adsorbed oxygen. On the other hand, Co_3_O_4_ requires high temperature for the detection of CO, so in the tested temperature range the response is low. The composite materials, on the other hand, show improved performance with respect to both base materials (see also Figure 13). All of the mixtures respond to CO with an increasing resistance. In particular, the best response characteristics are found for the 20% Co_3_O_4_ mixture, which has a faster response with respect to the base materials, a reduced optimum temperature (160 °C), and a larger response (0.8%/ppm @1000 ppm CO @160 °C). For all of the tested materials, the response at RT is negligible. Also, these tests show that the mixture with 70% Co_3_O_4_ has the smallest sensitivity to humidity.

## 7. Discussions and Conclusions

The experimental results that are presented in the previous sections can be summarized, as follows:

- even a small quantity of Co_3_O_4,_ has a major influence on the behavior of the sensing layer (reduction of the resistance in the presence of oxidizing gases);
- 50%–50% mixtures are characterized by a resistance that is lower than the resistance of both pure Al-ZnO and pure Co_3_O_4_;
- the behavior of the sensors cannot only be attributed to the presence of Co_3_O_4_ grains or Co_3_O_4_ grain paths, as it can be seen from the value of the baseline resistance, the activation energy in air, and the response to the tested gases. Both the magnitude of the response, the optimum temperature and the speed cannot be justified by the superimposition of the responses of the two base materials; and,
- water mainly influences the response of the base materials, especially to NO_2,_ and it causes an increase of the sensor responses; the tested composites show a reduced influence of water.

It is useful to underline that, from Equations (5), (6), and (14), it can be seen that the resistances describing the different junctions assume the same form: $R=R_{A}\exp\left(\frac{E_{A}}{k_{B}T}\right)$, all describing thermal activated conduction. Actually, for homo-junctions, the exponential terms are proportional to the Schottky barrier height, whereas for the hetero-junctions, this term depends on the ‘variation’ of the barrier height. Therefore, even if the barrier height in hetero-junctions, in the absence of gases (built-in potential), is large (approx. 1 eV), its variation is the factor determining the variation of the activation energy. Taking into account these considerations, the sensing material observed behaviors can be explained by the following hypotheses:

(1) hetero-junctions resistance is low;
(2) hetero-junctions respond to oxidizing gas (reducing gases) with a reduction of the activation energy and hence of the resistance (with an increase of the activation energy and hence of the resistance), therefore showing the same behavior as a p-type homojunction. Note that when in sensing layers there are hetero-junctions (between the n-type and p-type material) the behavior can’t be classified neither as p-type nor n-type.
(3) The resistance magnitudes of the three possible junctions are different, namely: at Al-ZnO–Al-ZnO boundaries, the resistance is high, at Co_3_O_4_ - Co_3_O_4_ is low, at Co_3_O_4_ –Al-ZnO is very low. Therefore, channels of Co_3_O_4_ grains or Co_3_O_4_-ZnO grain sequences result in the most relevant contributions to conduction.

To explain the influence of the water vapor, a complex model is required (see [59]) that discusses the water adsorption on SnO_2_. The water vapor must be treated in the same manner as the other gaseous chemical species, i.e., in mixture with air as a carrier, as a third chemical species added to the air oxygen and the target gas. Water dissociates and adsorbs at the surface, and it can react with pre-adsorbed and ionized oxygen. Differently from the usual assumption (used also in this paper in the modeling section, which describes the effect of chemisorbed molecules only through the surface depleted region variation, neglecting the influence on the bulk free carrier density) in the case of water chemisorption, also the variation of the number of free electrons (which can increase significantly) or holes (which can decrease significantly) should be taken into account, due to the large coverage that is expected. In this case, the reactions that are described in Equation (16) implying the ionization of NO_2_ can be significantly favored. Furthermore, the ionization of oxygen will be favored promoting the Equations (17a) and (17b). This can explain the behavior of pure n-type and p-type materials. The effect on the hetero-junction is more complex, in fact, by probably changing also the ‘bulk’ value of the free carriers on the two sides of the junction, the built-in potential can be changed, but on the n-side, the free carriers increase, whereas on the p-side, they decrease, so the two effects can compensate each other, as seems to be confirmed by the experimental results. 

In conclusion, the gas sensing properties of nanopowder that is composed of two different metallic oxides nanoparticles mixture are studied. The sensors are prepared by mixing Co_3_O_4_ (p-type semiconductor) and Al doped ZnO (n type semiconductor) nanoparticles with different ratios, varying from 0 to 100%. Since, in the mixtures, the grains that are in contact may form both homo-junctions and heterojunctions, a model of the sensor resistance taking into account these junctions is presented. The gas sensing testing towards a reducing gas, such as CO and oxidizing one, as NO_2_ reveals that the presence of heterojuctions remarkably improves and stabilizes the realized gas sensor sensitivity with respect to the base oxides. We noticed that 70% Co_3_O_4_ nano-powder exhibits better sensing properties towards NO_2_, while 20% Co_3_O_4_ nano-powder reveals better sensing towards CO. Additional measurements to assess the selectivity and in general the response to different oxidizing and reducing gases are ongoing. 

As a final remark, we stress that the composite behavior cannot be derived from those of the two base materials, but also that the behavior of a hetero-junction is quite complex. In fact, the chemisorption at the two sides of the junction is different, since it occurs on two different materials. In addition, the effect is the opposite at the two sides: on one side, the depleted region depth will increase and on the other one it will decrease. The overall effect on the barrier with respect to the undisturbed situation of no ionized charge trapped at the surface cannot be predicted unless the chemical reactions are completely known. In the studied case, for instance, in the presence of oxidizing gases, the effect that dominates is an overall reduction of the barrier height and a consequent reduction of the boundary resistance. Moreover, it should be added that one material can act as a catalyst for the adsorption on the other one, also favoring the adsorption at lower temperature or changing the adsorption kinetics, and this is in accordance with the experimental results that show, in general, faster responses for the composites.
